# A bendamustine resistance gene signature in diffuse large B-cell lymphoma and multiple myeloma

**DOI:** 10.20517/cdr.2020.76

**Published:** 2021-03-19

**Authors:** Issa Ismail Issa, Rasmus Froberg Brøndum, Hanne Due, Linnéa Schmidt, Martin Bøgsted, Karen Dybkær

**Affiliations:** ^1^Department of Haematology, Aalborg University Hospital, Aalborg 9000, Denmark.; ^2^Department of Clinical Medicine, Aalborg University, Aalborg 9000, Denmark.

**Keywords:** Bendamustine, diffuse large B-cell lymphoma, multiple myeloma, resistance gene signature

## Abstract

**Aim**: Bendamustine is primarily used for treatment of indolent lymphomas but has shown efficacy in some patients with diffuse large B-cell lymphoma (DLBCL) and multiple myeloma (MM). Molecular-based patient stratification for identification of resistant patients, who will benefit from alternative treatments, is important. The aim of this study was to develop a resistance gene signature (REGS) from bendamustine dose-response assays in cultures of DLBCL and MM cell lines, enabling prediction of bendamustine response in DLBCL and MM patients.

**Methods**: Bendamustine response was determined in 14 DLBCL and 11 MM cell lines. Using baseline gene expression profiles and degree of growth inhibition after bendamustine exposure, a bendamustine REGS was developed and examined for the risk stratification potential in DLBCL (*n* = 971) and MM (*n* = 1,126) patients divided into prognostic subtypes.

**Results**: Bendamustine resistance significantly correlated with resistance to cyclophosphamide in DLBCL and melphalan in MM cell lines. The bendamustine REGS showed significantly lower bendamustine resistance probabilities in DLBCL patients with GCB subtype tumors and in tumors of the differentiation dependent centrocyte and plasmablast subtypes. In MM patients, pre-BII classified tumors displayed high bendamustine resistance probabilities and the plasma cell subtype had lower bendamustine resistance probability than memory cells. Furthermore, tumors belonging to the 4p14, MAF, and D2 TC subclasses consistently displayed high bendamustine resistance probabilities.

**Conclusion**: Significant differences in predicted response to bendamustine were found in molecular subtypes of DLBCL and MM, encouraging validation in prospective bendamustine-treated cohorts with available gene expression profiles and follow-up data.

## Introduction

Bendamustine is a bi-functional drug used for treatment of chronic lymphocytic leukemia (CLL) and rituximab-refractory indolent non-Hodgkin’s lymphoma (NHL)^[1]^. It contains three structural components: (1) a nitrogen mustard (2-chloroethylamine group) which confers the alkylating function; (2) a benzimidazole ring (purine analog) giving it the antimetabolite function; and (3) a butyric acid group that confers water solubility^[2]^. The mechanisms of action of bendamustine include DNA cross-linking, activation of DNA damage response leading to apoptosis, inhibition of mitotic checkpoints, and induction of mitotic catastrophe - a form of non-apoptotic cell death^[3]^. Bendamustine is known to be well-tolerated and has also been shown to be effective as a single agent in heavily pretreated, high-grade NHL patients^[4]^.

The most common type of NHL is diffuse large B-cell lymphoma (DLBCL)^[5]^. DLBCL is an aggressive form of NHL and the standard treatment is immuno-chemotherapy consisting of rituximab, cyclophosphamide, doxorubicin, vincristine, and prednisone (R-CHOP). Unfortunately, up to one third of patients eventually develop refractory disease or relapse (R/R) in which case they are treated with high dose salvage therapy and autologous stem cell transplantations (ASCT)^[6]^. Not all diagnostic patients are eligible for full-dose R-CHOP due to toxicities which might lead to dose reduction, substitution, or discontinuation of one or more of the drugs^[7]^. Moreover, a substantial fraction of R/R patients are ineligible for ASCT due to comorbidities and age, and for these no standard salvage therapy exist^[6,8,9]^. Thus, effective alternative therapies with manageable toxicities are needed. In that context, bendamustine in combination with rituximab therapy has been suggested due to long-term remission for some patients together with low toxicity^[8,10,11]^. Several clinical studies have tested the effect of combined bendamustine and rituximab treatment in DLBCL patients and reported an acceptable toxicity profile with modest activity, meaning only a subset of the patients respond [Supplementary Table 1]^[4,12-18]^. Thus, strategies for patient stratification based on bendamustine response are needed.

Multiple myeloma (MM) accounts for up to 10% of all hematological malignancies^[19]^ and develops from post-germinal, antibody-secreting plasma cells that have undergone somatic hypermutation before they infiltrate the bone marrow^[20]^. Over the past decades, the survival of MM patients has improved drastically, mainly attributed by advances in novel therapies^[21,22]^. Melphalan combined with prednisone was the first successful therapy in achieving relatively high response rates in MM and has remained the gold standard for several decades. Of note, a phase III study has later revealed a generally superior outcome when using bendamustine in combination with prednisone in comparison to melphalan combined with prednisone, and it was thus approved as first-line therapy for MM patients not eligible for ASCT^[23]^. In R/R MM patients, the overall response to bendamustine monotherapy is 30%-55%^[24]^ and higher when combined with other chemotherapy agents [Supplementary Table 1]^[23,25-27]^. As with DLBCL, bendamustine combination treatment has been shown to be safe, effective, and well-tolerated in both diagnostic and R/R MM patients^[24,28,29]^.

Various prognostic molecular classifications have been developed based on gene expression profiles (GEPs) of DLBCL and MM cell lines and patient tumor samples. According to the 2016 revision of the World Health Organization classification of lymphoid malignancies, the standard molecular subclassification of DLBCL is activated B-cell-like (ABC) and germinal center B-cell-like (GCB) DLBCL which has proven to have prognostic impact favoring the GCB-DLBCL subtypes^[30,31]^. MM can be subclassified based on the translocation and cyclin D (TC) classification which distinguishes eight subtypes based on immunoglobin translocations, activation of cyclin D genes, and chromosomal ploidy^[32]^. A more refined cell-of-origin (COO) based classification system, the “B-cell associated gene signature” (BAGS), also exist for DLBCL and MM where primary cancer cells at the time of diagnosis are associated to normal B-cell subset phenotypes from tonsils or bone marrow, respectively. DLBCL patients are classified into one of the following subtypes: naïve, centrocyte, centroblast, memory B-cell, or plasmablast^[33]^, and MM patients into: Pre-BI, Pre-BII, immature, naïve, memory, or plasma cell^[34]^. BAGS provide for both DLBCL and MM independent and significant prognostic information as compared to ABC/GCB and TC, and for DLBCL when compared to the clinical international prognostic index. In addition, the BAGS subtypes have distinct and specific responses to chemotherapeutic agents used in a routine clinical setting for treatment of DLBCL and MM^[33,34]^. Machine learning models have previously been developed to predict response to components of the R-CHOP regimen in DLBCL and melphalan in MM. These models were successfully generated based on dose-response studies and resistance gene signatures (REGS) in human B-cell cancer cell lines (HBCCL)^[35-37]^.

In this study, we hypothesize that bendamustine dose-response assays and GEP of cell lines can be used to find gene expression patterns in which the expression of a group of genes predicts the cellular response to bendamustine. The predicted response can be applied to samples from DLBCL and MM patients to identify the subset of patients that will be sensitive or resistant to treatment. To test this, we developed a REGS from DLBCL and MM cell lines based on GEP and bendamustine dose-response assays, and subsequently investigated their stratification potential of DLBCL and MM patients based on molecular profiles.

## Methods

### Cell lines and identity confirmation

HBCCLs consisting of 14 DLBCL cell lines (DB, FARAGE, HBL-1, MC-116, NU-DHL-1, NU-DUL-1, OCI-Ly3, OCI-Ly7, OCI-Ly8, RIVA, SU-DHL-5, SU-DHL-8, SU-DHL-10, and U-2932) and 11 MM cell lines (AMO-1, KMS-11, KMS-12-BM, KMS-12-PE, LP-1, MOLP-2, MOLP-8, NCI-H929, OPM-2, RPMI-8226, and U266) were used. Culturing and DNA barcoding for verification of cell line identities were performed as previously described^[36,38]^. The cells were cultured at 37 °C in a humidified atmosphere of 95% air and 5% CO_2_ with the appropriate medium, serum, and supplements [Table 1].

**Table 1:** Information on cell lines used in this study^[34,38,39]^

Cell line	Disease	Culture media	ABC/GCB or TC classification	BAGS
DB	DLBCL	RPMI1640, 20%FBS	GCB	Centrocyte
FARAGE	DLBCL	RPMI1640, 10%FBS	GCB	Centrocyte
HBL-1	DLBCL	RPMI1640, 10%FBS	ABC	Centrocyte
MC-116	DLBCL	RPMI1640, 15%FBS	GCB	Centrocyte
NU-DHL-1	DLBCL	RPMI1640, 10%FBS	ABC	Memory
NU-DUL-1	DLBCL	RPMI1640, 15%FBS	UC	Naïve
OCI-Ly3	DLBCL	IMDM, 20%HS*	ABC	Centrocyte
OCI-Ly7	DLBCL	RPMI1640, 10%FBS	GCB	Centroblast
OCI-Ly8	DLBCL	RPMI1640, 10%FBS	UC	Centroblast
RIVA	DLBCL	RPMI1640, 10%FBS	ABC	Centrocyte
SU-DHL-10	DLBCL	RPMI1640, 10%FBS	GCB	Centrocyte
SU-DHL-5	DLBCL	RPMI1640, 20%FBS	GCB	Centrocyte
SU-DHL-8	DLBCL	RPMI1640, 10%FBS	GCB	Centroblast
U-2932	DLBCL	RPMI1640, 10%FBS	GCB	Centrocyte
AMO-1	MM	RPMI1640, 20%FBS	D2	Plasma cell
KMS-11	MM	RPMI1640, 10%FBS	4p16	Plasma cell
KMS-12-BM	MM	RPMI1640, 20%FBS	4p16	Plasma cell
KMS-12-PE	MM	RPMI1640, 20%FBS	4p16	Unclassified
LP-1	MM	IMDM, 10%FBS	4p16	Plasma cell
MOLP-2	MM	RPMI1640, 20%FBS	D2	NA
MOLP-8	MM	RPMI1640, 20%FBS	4p16	Plasma cell
NCI-H929	MM	RPMI1640, 10%FCS**	4p16	Plasma cell
OPM-2	MM	RPMI1640, 10%FBS	4p16	Plasma cell
RPMI-8226	MM	RPMI1640, 10%FCS	MAF	Naïve
U266	MM	RPMI1640, 10%FBS	4p16	Unclassified

Cell culture supplements: *2 mmol/L L-Glutamine, 55 µmol/L β-Mercaptoethanol; **2 mmol/L L-Glutamine, 1 mmol/L sodium pyrovate, 55 µmol/L β-Mercaptoethanol. FBS: fetal bovine serum; HS: human serum; GCB: germinal center B-cell-like; ABC: activated B-cell-like; TC: translocation and cyclin D; NA: not available

### Systematic dose-response assay

Prior to the dose-response experiments, the optimal seeding concentration for each cell line was determined as the highest concentration at which the cells grew at an exponential rate throughout the experiment without drugs added. The effect of bendamustine on viable proliferating cells was measured for the 25 HBCCL. Cells were seeded in 120 µL culture media per well in a 96 well plate 24 h before 16 bendamustine concentrations of two-fold increments were added. The highest bendamustine concentration used was 500 µg/mL in isotonic saltwater. Viability was measured immediately after addition of bendamustine (0 h plate) and after 48 h of drug exposure (48 h plate) by addition of CellTiter reagent (CellTiter 96 Aqueous One Solution Reagent, Promega, USA) and subsequent absorbance measurement at 492 nm using FLUOstar Optima (BMG LABTECH, Germany). Isotonic saltwater was used as controls alongside bendamustine. Border effects were circumvented by only including non-border wells for analysis for each experiment, and experiments were performed in triplicates. Time independent summary statistics were obtained as previously described^[36]^.

### Gene expression profiling datasets for cell lines and clinical cohorts

GEPs for each untreated cell line using the Affymetrix Human Genome U133 Plus 2.0 arrays were used. These were generated by purification of RNA from cells, cDNA conversion, biotin-labelling, and lastly hybridization to the GeneChip. The *.CEL* files for all cell lines are deposited with GEO accessions GSE53798^[35]^, GSE99634^[34]^, and GSE22759^[40]^. In DLBCL, three individual, diagnostic patient cohorts with available GEP datasets from tumors were used to test the bendamustine REGS, and GEP data were pre-processed as previously described^[41]^. These encompass the IDRC dataset (International DLBCL Rituximab-CHOP Consortium Program Study, *n* = 467 GEO accession: GSE31312)^[42]^, the LLMPP dataset (Lymphoma/Leukemia Molecular Profiling Project, with data divided based on subsequent treatment into LLMPPCHOP: *n* = 181 and LLMPPRCHOP: *n* = 233, GEO accession: GSE10846)^[43]^, and the MDFCI dataset (Mayo-Dana-Farber Cancer Institute, *n* = 90, GEO accession: GSE34171)^[44]^. Similarly, the following three separate and previously described^[34]^ MM patient cohorts were used to test the bendamustine REGS: HOVON-65 dataset (*n* = 320, GEO accession: GSE19784)^[45]^, MRC Myeloma IX dataset (*n* = 247, GEO accession: GSE15695, Platform: GPL570)^[46]^, and UAMS dataset (University of Arkansas for Medical Sciences, *n* = 559, GEO accession: GSE24080)^[47]^.

### Statistical analysis

For all statistical analyses R version 4.0.2 was used^[48]^. An Rmarkdown document detailing the analysis is available as Supplementary File 1.

Correlations between bendamustine, cyclophosphamide, doxorubicin, vincristine, and melphalan response were determined by assessing the pairwise Pearson’s correlation of the area under the positive parts of the dose-response curves (AUC_0_). In this aspect, previously generated summary statistics for the other drugs, used in the treatment of hematological cancers, were obtained in addition to the bendamustine dose-response assays^[35,40]^. A generalized pairs plot was created using the R package *GGally*^[49]^.

The bendamustine REGS is based on regularized multivariate regression analysis with cross-validated tuning parameters and can assign probability of resistance to a specific drug utilizing GEP and dose-response data from cell lines^[38]^. For the development of the bendamustine REGS, we followed a previously described approach^[35]^. Briefly, the HBCCL and clinical cohort microarray data were background corrected and Robust Multichip Average (RMA) pre-processed using the Bioconductor package *frma*^[50]^. Cell lines were split into tertiles according to their AUC_0_ values separately for each disease to avoid comparison of diseases and assigned values of sensitive, intermediate, or resistant. Sensitive and resistant cell lines were treated as binary outcomes and GEPs as predictors in a logistic regression model with an elastic net penalty using the R package *glmnet*^[51]^. Optimal parameters were obtained using leave-one-out cross-validation, with misclassification error as the loss function.

Cell lines and patient samples from cohorts were assigned to DLBCL or MM BAGS classes using the BAGS classifiers^[33,34]^. Assignment to a BAGS group was determined by the highest predicted probability across the classes, setting the 15% of patients with lowest classification probability in each cohort as unclassified. Kaplan-Meier curves of DLBCL patients were trichotomized based on predicted bendamustine response including p-values from a log-rank test generated using the R package *survminer*^[52]^.

## Results

### Bendamustine dose-response assay in a panel of 25 human B-cell cancer cell lines

Using the relative growth inhibition for a drug, it is possible to generate cell line specific dose-response curves^[38]^. However, faster growing cells generally appear more sensitive to chemotherapy drugs when using a fixed timepoint^[36]^, thus to account for this bias under the assumption of exponential growth, the growth rate from treated cells was normalized using untreated cells at both 0h and 48h time points as reference. This allows for a time independent determination of growth inhibition where growth rates are accounted for and hereby the bendamustine response among HBCCL panel can be ranked and compared. The four summary statistics from the drug response experiments are: 50% growth inhibition compared to untreated cells (GI_50_), total growth inhibition (TGI), the concentration at which the cell population is halved after 48 h (LC_48_), and AUC_0_ (the area under the curve above 0) [Table 2].

**Table 2:** Summary statistics and T_0_ from bendamustine dose-response assays consisting of: Doubling time for individual cell lines (T_0_) in hours, concentration to obtain GI_50_, TGI, 50% cell decay at 48 h (LC_48_), and area under the positive part of the dose-response curve (AUC_0_)

Cell line	T_0_	GI_50_	TGI	LC_48_	AUC_0_
AMO-1	30 (29;31)	1.84 (1.83;1.84)	1.91 (1.91;1.92)	2.01 (2.00;2.02)	350.77 (344.73;357.66)
DB	40 (39;41)	1.83 (1.83;1.84)	1.96 (1.95;1.97)	2.10 (2.10;2.10)	344.57 (338.43;347.85)
FARAGE	48 (46;50)	0.99 (0.96;1.01)	1.37 (1.34;1.39)	1.58 (1.57;1.59)	269.60 (258.59;273.48)
HBL-1	32 (31;32)	1.46 (1.44;1.47)	1.63 (1.62;1.64)	1.81 (1.80;1.82)	313.61 (308.36;316.95)
KMS-11	47 (45;49)	1.82 (1.81;1.82)	1.85 (1.85;1.85)	1.91 (1.91;1.92)	340.53 (330.90;348.95)
KMS-12-BM	38 (37;40)	1.30 (1.28;1.33)	1.53 (1.52;1.53)	1.62 (1.61;1.63)	287.42 (279.99;292.87)
KMS-12-PE	45 (43;47)	1.80 (1.80;1.80)	1.81 (1.81;1.82)	1.83 (1.82;1.84)	346.87 (336.46;354.13)
LP-1	37 (35;38)	2.07 (2.03;2.10)	2.12 (2.10;2.13)	2.16 (2.12;2.17)	351.59 (334.09;361.07)
MC-116	59 (55;62)	1.56 (1.52;1.57)	1.75 (1.71;1.78)	1.81 (1.81;1.81)	288.97 (274.71;294.63)
MOLP-2	129 (119;141)	0.99 (0.94;1.01)	1.20 (1.16;1.22)	1.80 (1.77;1.80)	250.41 (237.85;261.82)
MOLP-8	32 (31;33)	1.27 (1.25;1.28)	1.50 (1.47;1.52)	1.75 (1.72;1.78)	297.54 (293.42;302.04)
NCI-H929	43 (42;45)	1.80 (1.80;1.80)	1.83 (1.80;1.83)	1.88 (1.81;1.89)	340.65 (326.74;346.13)
NU-DHL-1	34 (33;35)	0.96 (0.93;0.99)	1.28 (1.27;1.29)	1.52 (1.51;1.52)	267.75 (261.68;269.21)
NU-DUL-1	43 (42;44)	0.60 (0.60;0.61)	0.73 (0.72;0.74)	0.96 (0.95;0.97)	228.13 (226.01;235.19)
OCI-Ly3	26 (25;26)	1.35 (1.33;1.36)	1.61 (1.57;1.64)	1.88 (1.87;1.89)	310.70 (304.25;314.61)
OCI-Ly7	44 (42;46)	1.54 (1.53;1.55)	1.69 (1.67;1.71)	1.83 (1.82;1.84)	269.17 (262.78;278.12)
OCI-Ly8	23 (23;24)	1.52 (1.49;1.54)	1.83 (1.82;1.83)	1.92 (1.91;1.93)	315.94 (309.48;320.47)
OPM-2	69 (66;73)	1.82 (1.81;1.83)	1.88 (1.87;1.89)	2.06 (2.05;2.07)	342.42 (329.41;350.07)
RIVA	40 (38;41)	1.34 (1.32;1.38)	1.56 (1.56;1.57)	1.73 (1.72;1.74)	286.65 (275.47;294.00)
RPMI-8226	43 (41;46)	2.10 (2.10;2.11)	2.10 (2.10;2.14)	2.11 (2.11;2.21)	378.98 (369.63;382.48)
SU-DHL-10	31 (30;32)	1.83 (1.83;1.83)	1.87 (1.87;1.88)	1.93 (1.92;1.93)	351.16 (343.86;357.56)
SU-DHL-5	35 (34;37)	0.44 (0.43;0.45)	0.62 (0.62;0.63)	0.78 (0.78;0.79)	219.79 (213.34;223.48)
SU-DHL-8	58 (56;61)	1.05 (1.03;1.07)	1.24 (1.22;1.26)	1.54 (1.52;1.56)	281.88 (269.61;283.06)
U266	58 (56;60)	1.80 (1.75;1.80)	1.88 (1.88;1.89)	2.09 (2.08;2.10)	333.48 (326.50;337.13)
U2932	46 (43;47)	1.16 (1.13;1.19)	1.37 (1.35;1.39)	1.68 (1.67;1.70)	288.84 (280.70;293.88)

All bendamustine concentrations are indicated as log10 (µg/mL), and confidence intervals are assigned using parametric bootstrap. GI_50_: 50% growth inhibition; TGI: total growth inhibition

**Figure 1:**
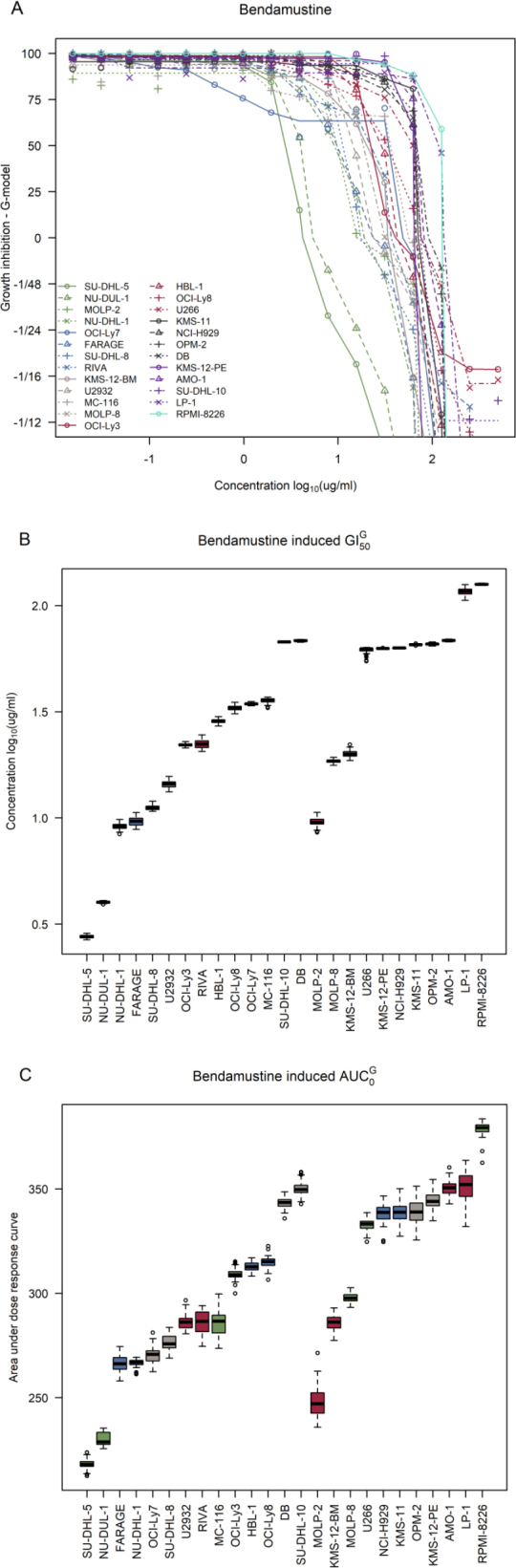
Dose-response curves for the bendamustine screens in DLBCL and MM cell lines. A: dose-response curves for the 14 DLBCL cell lines (DB, FARAGE, HBL-1, MC-116, NU-DHL-1, NU-DUL-1, OCI-Ly3, OCI-Ly7, OCI-Ly8, RIVA, SU-DHL-5, SU-DHL-8, SU-DHL-10, and U-2932) and 11 MM cell lines (AMO-1, KMS-11, KMS-12-BM, KMS-12-PE, LP-1, MOLP-2, MOLP-8, NCI-H929, OPM-2, RPMI-8226, and U266); B: ranked 50% growth inhibition (GI_50_) corrected for individual growth rates of the cell lines with DLBCL cell lines to the left and MM cell lines to the right; C: ranked area under the positive part of the curve (AUC_0_) values corrected for individual growth rates of the cell lines with DLBCL cell lines to the left and MM cell lines to the right. DLBCL: diffuse large B-cell lymphoma; MM: multiple myeloma

Increasing concentrations of bendamustine induced growth inhibition at varying degrees for both DLBCL and MM cell lines [Figure 1A]. Cell decay can be seen in the bottom half of the dose-response curves which show half times (e.g., a value of -1/48 is the concentration at which the cell population is halved after 48 h). Overall, DLBCL cell lines were less resistant to bendamustine than MM cell lines [Figure 1B and C] with AUC_0_ ranging from 219.79 (95%CI: 213.3; 223.5) to 351.16 (CI: 343.9; 357.6) in DLBCL and 250.41 (CI: 237.9; 261.8) to 378.98 (CI: 369.6; 382.5) in MM cell lines. The most sensitive cells were SU-DHL-5 and MOLP-2 in DLBCL and MM, respectively, and the most resistant were SU-DHL-10 and RPMI-8226, respectively.

Interestingly, the six MM cell lines, U266, KMS-12-PE, NCI-H929, KMS-11, OPM-2, and AMO-1 displayed very similar GI_50_ and AUC_0_ values with GI_50_ ranging from 1.80 (CI: 1.75; 1.80) to 1.84 (CI: 1.83; 1.84) and AUC_0_ ranging from 333.5 (CI: 326.50; 337.13) to 350.8 (CI: 344.7; 357.7) [Figure 1B and C]. Characteristically, these six cell lines belong to the plasma cell or unclassified BAGS-MM subtypes, and five of the cell lines belong to the TC 4p16 class whereas AMO-1 is TC D2 [Table 1]. RPMI-8226 is noticeably more resistant than the rest of the MM cell lines and has a unique profile as it is BAGS classified as naïve with a MAF TC subclass. For DLBCL, most cell lines were either BAGS classified as centroblasts or centrocytes.

### Assessment of correlations in response to other hematological drugs

To investigate co-occurrence of resistance and sensitivity among the HBCCL towards various drugs used in the treatment of hematological cancers, we assessed the pairwise Pearson’s correlation between AUC_0_ values for bendamustine from this study and the cornerstone drugs of standard DLBCL and MM treatment: cyclophosphamide, doxorubicin, vincristine, and melphalan obtained from previous dose-response assays [Supplementary Table 2]^[35,40]^. A significant and positive correlation was regarded as an indication of shared resistance mechanisms between two compared drugs.

**Figure 2:**
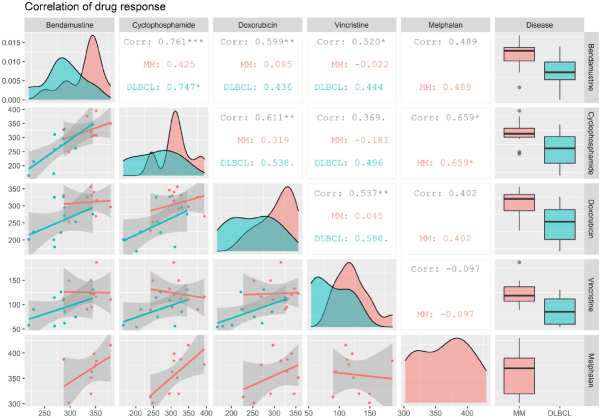
Correlations between drug response in HBCCL. A generalized pairs plot, showing correlations in response between two drugs in DLBCL and MM cell lines. Distributions and plots are based on values for area under the positive part of the curve (AUC_0_) which are displayed on the axes, and the shaded area shows the 95% confidence interval. Data for melphalan in DLBCL was not included since there were too few melphalan treated cell lines available. Corr: correlation. _•_*P* < 0.1, *P < 0.05, **P < 0.01, ***P < 0.001. DLBCL: diffuse large B-cell lymphoma; MM: multiple myeloma; HBCCL: human B-cell cancer cell lines

In DLBCL cells, the highest and only significant correlation coefficient was found between bendamustine and cyclophosphamide treatment (*R* = 0.747, *P* < 0.05) [Figure 2]. Moderate and non-significant correlations in response were also observed for bendamustine compared to doxorubicin and vincristine, both of which displayed the lowest correlation among all the drugs in DLBCL cell lines (*R* = 0.44 for both). Melphalan dose-response data was not included for DLBCL cell lines since there were too few treated cell lines for comparison, and it is not widely used in the treatment of DLBCL. In MM, correlation coefficients between the drugs were overall lower [Figure 2]. The strongest, albeit non-significant, correlation for bendamustine was found in comparison to melphalan (*R* = 0.49) and cyclophosphamide (*R* = 0.43) - two other alkylating agents. Interestingly, these two drugs displayed the overall strongest and most significant correlation in MM (*R* = 0.659, *P* < 0.05) [Figure 2]. The lowest drug response correlation in MM was observed for vincristine, which exerts its mechanism of action during the M-phase in contrast to the other four drugs. In addition, there was a very low correlation between bendamustine and doxorubicin response in MM cell lines (*R* = 0.085). In summary, a stronger correlation in response between agents with the similar mechanisms of action was observed, indicating that there are inherent response patterns to similar drugs in the HBCCL.

### Stratification of clinical datasets based on predicted bendamustine response

A combination of GEPs from untreated cells in the HBCCL panel and outcome from the bendamustine dose-response assays was used to develop the bendamustine REGS. The minimum misclassification error in the cross-validation was 0.33. The final prediction model trained on the complete dataset, using the optimal parameters obtained from the cross-validation, generated a bendamustine REGS with 13 genes (*CCND2, EVI2A, HLA-DRA, SERPINF1, HMGN3, COX7A2, CD52, JCHAIN, HSPA1A, MPEG1, PRKAR2B, CLEC2B* and *QPCT*) [Supplementary Table 3]. Both DLBCL and MM tumors display molecular heterogeneity whereby cells from these diseases can be subclassified into different subtypes showing different prognosis after treatment^[53,54]^. Thus, we evaluated whether there was a difference in the predicted bendamustine response using the bendamustine REGS on GEPs restricted to DLBCL and MM patients and on different subtypes hereof [Supplementary Table 4].

**Figure 3:**
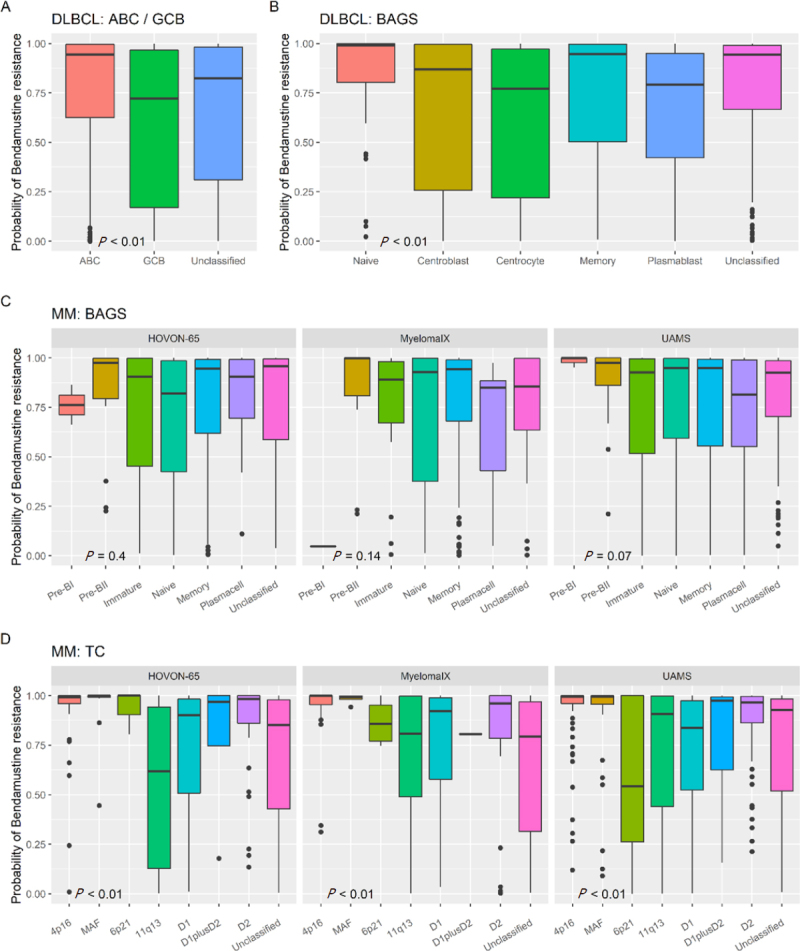
Bendamustine resistance probability in COO subtypes of patient cohorts. A: bendamustine REGS in ABC/GCB subtypes of DLBCL; B: bendamustine resistance probabilities using the bendamustine REGS in BAGS-DLBCL subtypes; C: bendamustine resistance probability using the REGS in BAGS-MM subtypes separately for each dataset; D: bendamustine resistance probability using the REGS in TC subclasses separately for each dataset. Significant differences between subtypes were tested using Kruskal-Wallis tests. DLBCL: diffuse large B-cell lymphoma; MM: multiple myeloma; BAGS: B-cell associated gene signature; REGS: resistance gene signature; COO: cell-of-origin; ABC: activated B-cell-like; GCB: germinal center B-cell-like

In DLBCL, the bendamustine REGS was tested in four independent, diagnostic DLBCL cohorts. As there was no significant difference in bendamustine resistance probabilities between the four cohorts [Supplementary Figure 1A], they were merged into one large cohort for further analyses. Subclassification into ABC/GCB subtypes showed there was a significant difference in predicted bendamustine response (*P* < 0.01), where ABC patients displayed the highest bendamustine resistance probability in contrast to GCB patients [Figure 3A]. When the merged DLBCL cohorts was divided into BAGS subtypes, a significant difference between the molecular subtypes was observed (*P* < 0.01) despite the large variations in resistance probability. The DLBCL patients with reminiscent transcriptional expression patterns matching centrocyte and plasmablasts displayed lower probabilities of bendamustine resistance in comparison to the naïve, centroblast, memory, and unclassified subtypes [Figure 3B]. Analysis of the BAGS subtypes separately for patients belonging to the ABC and GCB subtypes showed significant differences in predicted response to bendamustine in molecular subtypes reflecting the degree of cellular differentiation in both groups [Supplementary Figure 1C and D]. Of notice, tumors of the memory subtype in ABC-DLBCL patients displayed high probabilities of bendamustine resistance, whereas they were predicted to be the least bendamustine resistant subtype along with plasmablasts in the GCB-DLBCL patients.

For the MM cohorts there was a significant difference in the distribution of bendamustine resistance probabilities between the three datasets used [Supplementary Figure 1B]. Thus, subsequent comparison to the BAGS classification was performed in each individual dataset [Figure 3C]. When the bendamustine REGS was applied to the MM datasets, we did not find any significant difference between BAGS subtypes, however, the plasma cell subtype had the lowest bendamustine resistance probability in the MyelomaIX and UAMS datasets and was less resistant than the memory subtype in general. Additionally, the Pre-BII subtype had a high bendamustine resistance probability in all datasets [Figure 3C, Supplementary Figure 1E]. Significant differences between the TC subclasses were found in all three datasets, where the highest bendamustine resistance probability was consistently found for the TC classes: 4p14, MAF, and D2 [Figure 3D].

We did not find any bendamustine treated DLBCL cohorts usable for retrospective validation of the prediction model. However, as there was a significant correlation between bendamustine and cyclophosphamide response in the HBCCL *in vitro*, the predictive value of the bendamustine REGS was evaluated in patients treated with cyclophosphamide containing drug regimens. The LLMPP clinical DLBCL dataset, which is split into CHOP and R-CHOP treated patients, was used, and patients were trichotomized into sensitive, intermediate, and resistant groups based on predicted bendamustine response. Bendamustine resistance probabilities using the bendamustine REGS were significantly associated with overall survival in the CHOP treated cohort (*P* < 0.01), but not in the R-CHOP treated cohort [Supplementary Figure 2A and B]. However, when the bendamustine REGS and a previously generated cyclophosphamide REGS was compared in patients within each of the clinical cohorts, no significant correlation was found between the two REGSs [Supplementary Figure 3].

In summary, in clinical samples (DLBCL: *n* = 971, MM: *n* = 1,126) with individually assigned bendamustine resistance probabilities, we found that the ABC subtype was the most resistant in DLBCL, and the centrocyte and plasmablast subtypes were predicted to be the least resistant in the DLBCL patient cohorts. Bendamustine resistance probabilities in memory subtype tumors depended on ABC/GCB subclassification. In MM, the Pre-BII subtype had a high bendamustine resistance probability along with TC classes 4p14, MAF, and D2. Thus, molecular and COO based subclassification of patient cohorts can stratify patients based on bendamustine resistance probabilities.

## Discussion

Bendamustine is a good choice for treatment of elderly, frail, comorbid, or transplant ineligible DLBCL and MM patients, however, a fraction of these patients do not respond with sufficient efficacy^[24,55,56]^, urging the development of patient stratification tools for better prediction of responders and non-responders. Both DLBCL and MM display great molecular heterogeneity^[53,54]^ which might play a role in the response to bendamustine. The aim of the current study was to develop bioinformatics algorithms, enabling the prediction of bendamustine response in DLBCL and MM patients based on a combination of *in vitro* bendamustine dose-response assays in DLBCL and MM cell lines and their baseline GEPs. Within the HBCCL panel, response to bendamustine did not correlate with subtypes of BAGS nor with ABC/GCB subtypes for DLBCL. For MM, most of the cell lines were TC classified as 4p16 whereas the most bendamustine resistant cell line, RPMI-8226, was classified as MAF.

Bendamustine contains both a 2-chloroethylamine group also found in cyclophosphamide, melphalan, and chlorambucil, which confers the alkylating properties and a purine-like benzimidazole ring, which is found in nucleoside analogs, giving it an antimetabolite function^[2]^. Significant correlation between response to bendamustine and cyclophosphamide was observed in DLBCL cell lines and between melphalan and cyclophosphamide in MM cell lines. In addition, there was a moderate correlation between bendamustine response with cyclophosphamide and melphalan in MM cell lines. Higher coefficients of correlation suggest that the agents have similar mechanisms of action which is to be expected as these are all alkylating agents. Strong correlations have previously been found between cyclophosphamide, melphalan, and chlorambucil in the NCI-60 pan cancer cell panel, however, correlation between these agents and bendamustine was not as strong^[3]^. As expected, the similar mechanisms of action support the cross-resistance observed between bendamustine and chlorambucil and also to the nucleoside analog, fludarabine, in cells from CLL patients^[57]^.

Bendamustine has synergistic effects in DLBCL and MM cell lines when combined with various nucleoside analogs and other alkylating agents such as cyclophosphamide, chlorambucil, and melphalan, mainly due to its purine analog-like properties^[58]^. Synergistic effects with nucleoside analogs, but not with chlorambucil, was also shown in CLL cell lines^[57]^. These results indicate that bendamustine can be combined with other alkylating agents and purine analogs due to its bi-functional property and unique mechanisms of action. Moreover, synergy might not be necessary to achieve curative drug combinations exemplified by the antagonistic interaction seen by vincristine in combination with cyclophosphamide and doxorubicin at higher concentrations in DLBCL cell lines, where the combinations were still able to induce cell death due to non-overlapping resistance mechanisms^[59]^.

Bendamustine REGS in patients assigned to the phenotypic BAGS groups showed that patients belonging to the centrocyte and plasmablast subtypes in DLBCL, and the plasma cell subtype in the MyelomaIX and UAMS MM cohorts were predicted to be less resistant to bendamustine than the rest. Additionally, the bendamustine resistance probability was lower in the GCB-DLBCL subtype than in the ABC-DLBCL subtype. Thus, in a clinical setting, patients with a reminiscent transcriptional profile of these subtypes are suggested to be more sensitive towards bendamustine.

The use of malignant HBCCLs *in vitro* have limitations when compared to *in vivo* models, including scarce information on the tumor microenvironment and interaction with the immune system, lack of pharmacokinetic information (e.g., metabolism) regarding the drugs before reaching the tumor, and lack of consideration for intra- and inter-patient clonal heterogeneity of tumors. Despite these limitations, malignant cancer cell lines, like the HBCCLs, serve as strong pre-clinical models for disease specific pharmacogenomic studies (e.g., discovery of resistance genes, new drug targets, combinatorial effects, and cross-resistance) as they harbor many of the same mutations and genomic alterations found in malignant B-cells from DLBCL and MM^[60,61]^. In addition, the HBCCLs were originally developed from patients and display many of the cytogenic characteristics of DLBCL and MM which contribute to oncogenesis and possibly response to treatments^[61]^. The translational advantage of the HBCCL is the number of DLBCL and MM tumor specific cell lines in contrast to other cell panels such as NCI-60, which only contains two DLBCL and MM like cell lines.

The prognostic potential in patient stratification using the cell line based bendamustine REGS should be validated in large, independent, and prospective randomized clinical cohorts treated with or without bendamustine in combination with standard treatment regimens to assess its clinical potential. At the current time and to our knowledge, no bendamustine treated DLBCL and MM validation cohorts with available GEP data and follow-up exist. We have previously validated a melphalan REGS, where high probabilities of resistance were associated with inferior clinical outcome in the HOVON-65 dataset^[37]^. Furthermore, a combined REGS developed from cyclophosphamide, doxorubicin, and vincristine treated cell lines also showed prognostic value in DLBCL^[35]^.

In addition to the correlation between cyclophosphamide and bendamustine response *in vitro*, significant differences were found in the overall survival between patients trichotomized based on predicted bendamustine resistance in the CHOP treated cohort. This could suggest that the bendamustine REGS is a pseudo marker for response to treatment with alkylating agents sharing similar mechanisms of action with bendamustine in DLBCL. It is, however, worth noting that the DLBCL patients from the clinical datasets were subjected to a combination treatment containing either four or five drugs with different mechanisms of actions, some of which both interact with each other and the surrounding tumor microenvironment affecting the clinical outcome^[59,62]^. Additionally, the bendamustine resistance probabilities did not correlate with the cyclophosphamide resistance probabilities in patients. Thus, a bendamustine treated cohort is needed for validation of the bendamustine REGS. Strong and validated predictors of treatment outcome based on clear molecular signatures have a great potential in the clinical setting for the identification of resistant patients who will benefit more from alternative treatments. However, many biological and statistical considerations are needed prior to their development and clinical application.

In conclusion, the use of disease-specific cell lines is a strong tool for prognostic classification of patient cohorts as they can be used to create REGSs which can be further applied to clinical samples and used in risk stratification, either alone or in combination with other molecular subclassification models. Here, we evaluated the REGS for bendamustine in COO based subtypes in DLBCL and MM. For DLBCL, we found that the GCB subtype and the differentiation dependent BAGS subtypes of centrocyte, plasmablasts are predicted to have lower bendamustine resistance along with the plasma cell subtype in MM. However, these findings need to be validated in large, independent, prospective patient cohorts with bendamustine-treated patients and with available GEP and follow-up.

## Declarations

### Availability of data and materials

The datasets used in this article can be accessed through the National Center for Biotechnology Information Gene Expression Omnibus (https://www.ncbi.nlm.nih.gov/geo).

### Copyright

© The Author(s) 2021.
